# Effects of Anion Coadsorption on the Self-Assembly of 11-Acryloylamino Undecanoic Acid on an Au(111) Electrode

**DOI:** 10.1021/acsomega.4c05080

**Published:** 2024-09-10

**Authors:** Yi-Ting Huang, Jia-Yin Chen, Chiao-An Hsieh, Yamuna Ezhumalai, Chun-Jen Huang, Shuehlin Yau

**Affiliations:** † Department of Chemistry, National Central University, Chungli County, Taoyuan City 32049, Taiwan ROC; ‡ Department of Chemical and Materials Engineering, National Central University, Chungli County, Taoyuan City 32049, Taiwan ROC; § R&D Center for Membrane Technology, Chung Yuan Christian University, 200 Chung Pei Rd., Chungli County, Taoyuan City 32023, Taiwan ROC

## Abstract

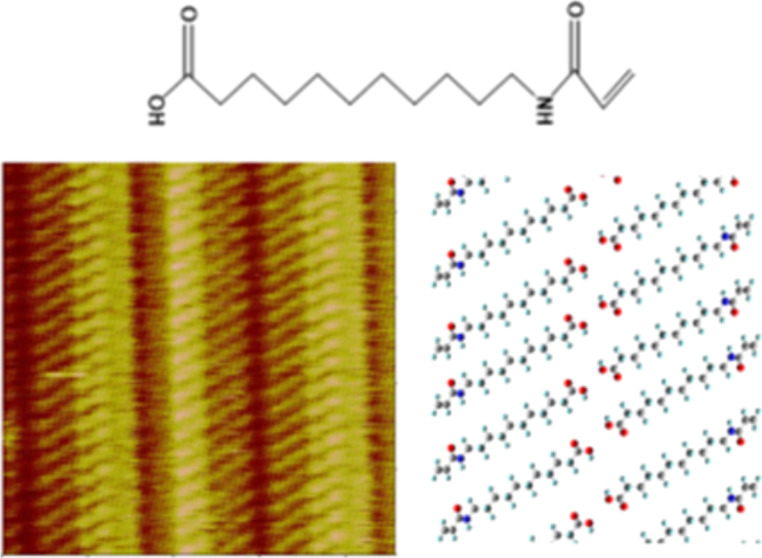

11-acryloylamino undecanoic acid (AAUA) is a versatile polymerizable surfactant that has been applied to coat medical devices, and these applications can benefit from a fundamental understanding of its interaction with a metal substrate. Cyclic voltammetry and *in situ* scanning tunneling microscopy (STM) were used to examine the adsorption configuration of AAUA molecules on an ordered Au(111) electrode and their mutual interactions, as AAUA was adsorbed from a methanol dosing solution. In addition to the van der Waals force between the aliphatic groups, the hydrogen bonding between the carboxylic acid and acrylamide groups was also important to guide the spatial arrangement of AAUA admolecules on the Au electrode. The −COOH group of AAUA admolecule likely dissociated in neutral media to −COO^–^, which formed hydrogen bonds with H_2_PO_4_^–^ in phosphate buffer solution (PBS). This interaction between the AAUA admolecules and ions in the electrolyte resulted in different electrochemical characteristics observed in phosphate buffer solution (PBS) and potassium sulfate (K_2_SO_4_). Molecular-resolution STM imaging revealed distinctly different AAUA spatial structures on the Au electrode in PBS and K_2_SO_4_. Shifting the potential positively to 0.5 V (versus Ag/AgCl) led to lifting of the reconstructed Au(111) to the (1 × 1) phase and the dissolution of the ordered AAUA film, suggesting that the orientation of the AAUA admolecule was altered. The ordered AAUA adlayer could be partially recovered by shifting the potential negatively.

## 1 Introduction

**Scheme 1:**
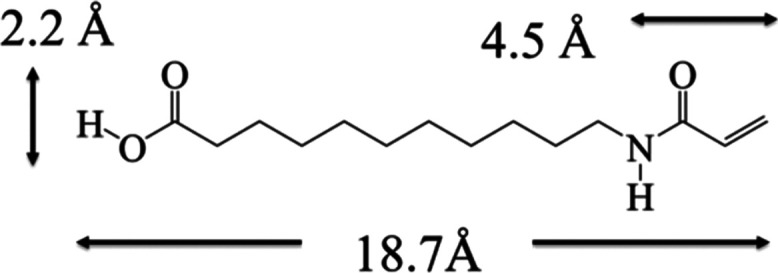
Molecular Structure of AAUA

11-acrylaminoundecanoic acid (AAUA), a polymerizable surfactant, has a reactive acrylamido group and fatty acid with a secondary amide linkage at the end of the hydrocarbon chain. The molecular structure of AAUA is shown in Scheme 1. The critical micelle concentration (CMC) of AAUA is so low that it can spontaneously self-assemble in a stable ordered form in a dilute aqueous solution.^1^

Many diverse and significant applications of AAUA have been reported. It can be used as the stationary phase to separate neutral and polar solutes in the capillary electrochromatography.^2^ AAUA-based polyelectrolytes can enhance the floc size, settling rate, and water quality, similar to the commercial coagulants used in wastewater treatment.^3^ Moreover, AAUA can copolymerize with zwitterionic and anionic monomers *via* controlled radical polymerization, yielding materials that are responsive to environmental stimuli and the subsequent formation of spherical polyion complex micelles and vesicles. This feature is useful in controlling drug release.^4,5^ AAUA can link polymeric material and metal,^6^ generating a polymer–metal hybrid as a dynamic device suitable for complex environments.^7,8^ A fundamental understanding of the interaction between AAUA and metal would be beneficial to the research of the polymer–metal hydrid.^7,9^

In the current study, an *in situ* scanning tunneling microscope (STM) was used to probe the adsorption of AAUA on an electrified Au(111) interface in phosphate buffer solution (PBS) and potassium sulfate (K_2_SO_4_). The importance of the alkane chain of adsorbate in guiding its interaction with others or with the Au(111) substrate has been investigated by STM imaging in organic solvent,^10−12^ in vacuum,^13,14^ and electrochemistry.^15^ Since alkane molecules interact with an Au electrode *via* the van der Waals force, it is likely that the charge density of an electrode would affect their strengths of adsorption.

Moreover, the AAUA molecule has a carboxylic group (−COOH) and an amide group (−NHCO−), which can form hydrogen bonds with other species to produce large molecular frameworks on the Au(111) electrode, as illustrated by others.^16−20^ The uses of these functional groups to tailor complex molecular organizations at the interface have been reported.^21−23^ For example, the amide group enables a bead–thread assembly, leading to a supramolecular network.^24^ Two adsorbates with and without an amide entity in their molecular structures are readily separated on a substrate.^22^ More recently, it is shown that the STM tip was used to trigger local protonation of terephathalic acid adsorbed on the Ag(111) substrate.^25^

The current STM study explored AAUA molecules adsorbed on the electrified Au(111) interface in PBS and K_2_SO_4_, revealing the important role of anions in building an ordered AAUA adlayer. Real-time STM scanning with molecular resolution was achieved to provide a direct view of the effects of potential modulation on the AAUA/Au(111) interface.

## 2 Experimental Section

AAUA was synthesized by reacting 11-aminoundecanoic acid with acryloyl chloride in a mixed ethanol/H_2_O (250/25 mL) solution. 11-aminoundecanoic acid (0.7623 mol) was added first, followed by NaOH (0.15 mol) until the solution became clear, and finally, acryloyl chloride (0.0743 mol) was added dropwise. The mixture was heated and stirred at 100 °C for 3 h. After filtration, the filtrate was acidified with diluted hydrochloric acid and washed with deionized water. A white precipitate was collected.^2^

The Au(111) electrodes used for voltammetry and STM experiments were single crystal beads made by melting the end of a poly gold wire using a hydrogen torch, followed by cutting and polishing to mirror finish with Al_2_O_3_ powder from 5 to 0.5 μm. The Au(111) electrode was pretreated with the conventional annealing and quenching method, resulting in the reconstructed Au(111)-(√3 × 22) structure.^26−28^

After being removed from the quenching tube, this Au(111) crystal was rinsed with acetone and placed in an AAUA/methanol dosing solution. It was then blown dry with a nitrogen stream and mounted onto an STM stage. The subsequent STM imaging reveals that this method produced an ordered AAUA adlayer on the Au(111) electrode, regardless of [AAUA] between 0.1 to 10 mM. The dosing time was fixed at 1 min, which was sufficient to have a full monolayer of AAUA.

Phosphate buffer solution (PBS) was prepared by mixing Na_2_HPO_4_ (J.T. Baker, Tokyo, Japan) and NaH_2_PO_4_ (Merck, Darmstadt, DFG). Potassium perchlorate and sulfate were purchased from Fisher Chemicals (Pennsylvania) and Showa Chemicals (Tokyo, Japan). These chemicals were used without further purification. Triple-distilled water (Lotun Technology Co., Taipei) was used to prepare all of the electrolytes.

Voltammetry experiments were performed in an electrochemical cell equipped with an Ag/AgCl reference electrode and a Pt counter electrode. The Au(111) electrode formed a hanging meniscus with the electrolyte. All measurements were performed at room temperature. The potentiostat was a CH 625 instrument (CH Instruments, Austin, TX).

The STM was produced by Veeco (Santa Barbara, CA) and equipped with a high-resolution scanner with a maximal scan size of 500 nm. The tip was a tungsten tip etched by AC in 1 M KOH and coated with Apeazon wax for insulating purposes. The optimal potential of the tip electrode was 0.3 V, which resulted in the lowest leakage current. The set point current was mostly lower than 1 nA. All STM images presented in this report were acquired with the constant current mode. The scan rates were 12 and 60 Hz for gross and fine scales of STM scanning.

All STM imaging experiments were conducted in a solution that was open to the air. Thermal drift in the piezo scanner was inevitable, which caused errors (±5%) in the lateral distance and vertical corrugation height. This “*in situ* STM” approach allowed us to examine the effects of potential control on the adsorption of AAUA molecules and anions on the Au electrode without the need to remove the sample from the electrolyte.^26,29−31^

## 3 Results and Discussion

Cyclic voltammetry (CV) is an electrochemical technique capable of detecting changes at the electrified interface in a submonolayer level and was used to have a quick survey of the process occurring at the AAUA-modified Au(111) electrode. The observed CV features were then substantiated with *in situ* STM imaging. The inevitable anions could interact with the admolecules and Au electrode to different extents, leading to dissimilar interfacial structures. This anionic effect is relevant to the practical use of AAUA as a medical coating because anions such as phosphate and chloride are commonly found under physiological conditions.

### 3.1 Cyclic Voltammetry

**Figure 1:**
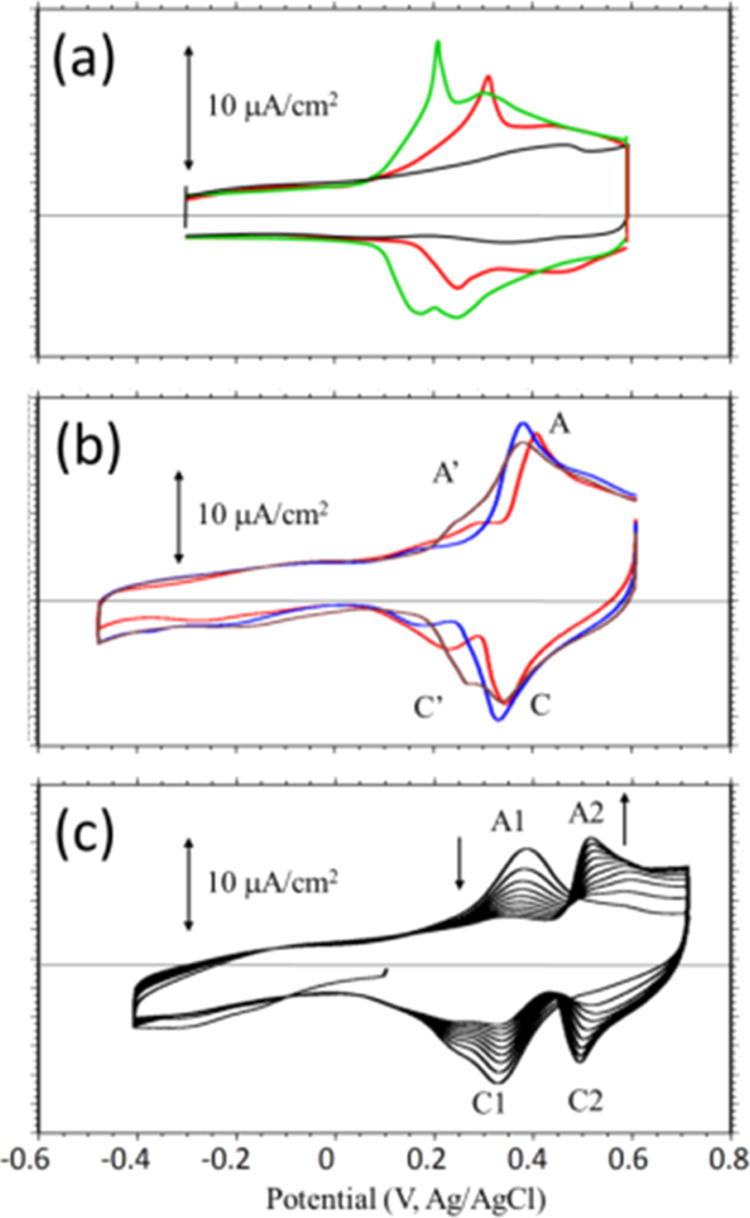
CVs recorded at 50 mV/s with bare Au(111) in 0.1 M PBS (green), KClO_4_ (black), and K_2_SO_4_ (red) (a), with the AAUA-modified Au(111) electrode in 0.1 M PBS (b) and K_2_SO_4_ (c). The dosing [AAUA] = 0.1 (brown), 1 (blue), and 10 mM (red) in panel (b).

The CVs recorded at 50 mV/s with a bare Au(111) electrode in 0.1 M PBS, KClO_4_, and K_2_SO_4_ are shown in green, black, and red lines in Figure 1a. The positive-going potential sweep in PBS (K_2_SO_4_) resulted in a notable peak at 0.2 V (0.32 V) but a mostly featureless profile in KClO_4_. These features are associated with anion adsorption on the Au(111) electrode and the coupled phase transition from the reconstructed Au(111)-(√3 × 22) to (1 × 1) at *E* > 0.1 V. The strengths of adsorption of these anions can be inferred from the peak potentials. The more negative the peak potential, the more strongly the anions were adsorbed. This view leads to a descending trend of anion adsorption on the Au(111) electrode, H_2_PO_4_^–^ or HPO_4_^2–^ > SO_4_^2–^ > ClO_4_^–^. Our STM results (not shown) reveal none of these anions formed an ordered structure on Au(111), as found with the Au(111) electrode in pH 4 sulfate and phosphate media.^32^

The AAUA-modified Au(111) sample was first examined in 0.1 M PBS. Three Au samples were prepared by immersion in the AAUA/methanol dosing solutions with [AAUA] of 10, 1, and 0.1 mM for 1 min. The obtained CVs are shown in red, blue, and brown lines in Figure 1b. The positive-going potential sweeps with these samples at 50 mV/s from −0.5 to 0.6 V resulted in a major peak at ∼0.4 V (A) and a minor shoulder (A′) at 0.25 V. Their counter features in the negative-going potential sweep emerged at 0.32 V (C) and 0.25 V (C′).

Evidently, these profiles have distinctively different morphologies from the background CV (green line in Figure 1a), indicating that the AAUA molecule was irreversibly adsorbed on the Au(111) electrode from these dosing solutions. Aided by STM results (described below), we ascribe the main peak A to the restructuring of the AAUA adlayer and the coadsorption of anion. The reconstructed Au(111) surface was also lifted to the (1 × 1) phase. Compared with that of a bare Au(111), the main peak A seen with the AAUA-modified Au(111) is 0.2 V more positive, indicating that the reconstructed Au(111) structure was stabilized by AAUA admolecules, as indeed seen with the STM.

In addition to the main peaks of C and C′, a minor feature emerged at −0.2 V in the negative-going potential sweep, as the AAUA adlayer underwent an order-to-disorder transition and was slowly desorbed. In contrast with hydrophobic admolecules, the current AAUA has hydrophilic groups of amide and −COO^–^ in its molecular structure, which resulted in a larger charging current flowing between −0.4 and 0.1 V.

The CV profiles obtained with AAUA-modified Au(111) in 0.1 M K_2_SO_4_ are shown in Figure 1c. As the potential was cycled between −0.4 and 0.7 V, a pair of broad peaks emerged at 0.35 V (A1/C1) initially but was gradually displaced by a new, reversible feature at 0.5 V (A2/C2). These CV variations are largely associated with anion coadsorption at *E* > 0.1 V, leading to the restructuring of the AAUA adlayer on the Au electrode, as evidenced by STM imaging (see below).

Moreover, these CV profiles recorded in PBS and K_2_SO_4_ have notably different morphologies, indicating that AAUA interacts differently with the anions in these electrolytes. In particular, one notes the possibility of hydrogen bond formation with H_2_PO_4_^–^ or HPO_4_^2–^ in PBS but not with SO_4_^2–^ in K_2_SO_4_.

### 3.2 *In Situ* STM

**Figure 2:**
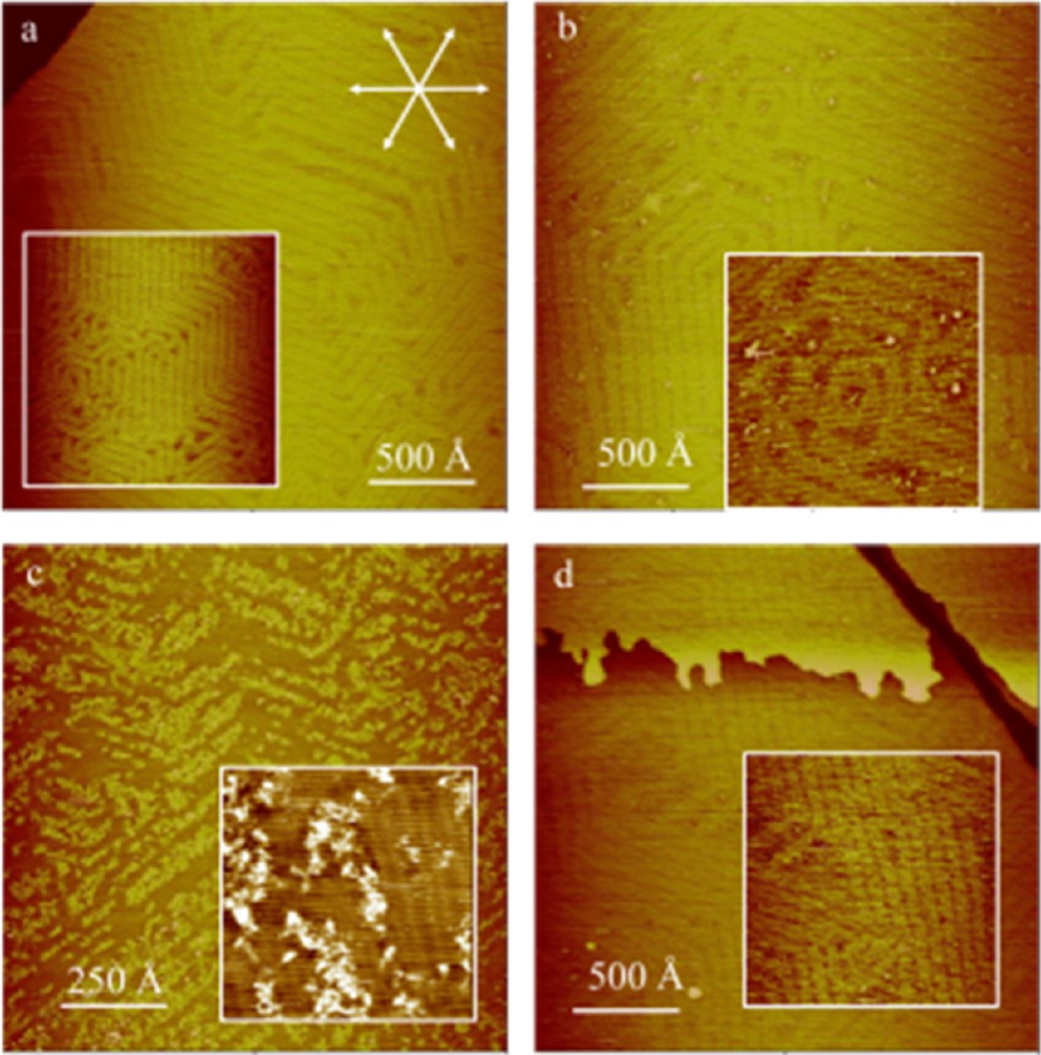
*In situ* STM images showing changes at a bare Au(111) electrode with the potential shifted from −0.1 (a) to 0.05 (b), 0.3 (c), and back to −0.1 V (d) in PBS. The inset in each panel reveals a finer resolution scan. Adsorption of HPO_4_^2–^ on the Au electrode was seen initially in panel (b), became prominent in panel (c), and was removed in panel (d). The elongated linear patterns define the close-packed atomic direction of the Au(111) electrode, shown as arrows in panel (a).

The interface of a bare Au(111) electrode was first examined with *in situ* STM in 0.1 M PBS. The ideal reconstructed Au(111)–(√3 × 22) surface was imaged at −0.1 V, as shown in Figure 2a. Switching the potential positively resulted in no change in the reconstructed Au(111) surface until 0.05 V, where random patches appeared (Figure 2b). These features are ascribed to adsorbed anions (such as HPO_4_^2–^), whose population increased with positive potential, as revealed by Figure 2c, collected at 0.3 V. The specifically adsorbed anion bulged by *ca.* 1.3 Å from the smooth Au background.

A close examination of the finer resolution STM scan shown in the inset of Figure 2c reveals that anions were preferably adsorbed at the fcc domain of the reconstructed Au(111) surface, with the (1 × 1) phase being installed at some local areas. These electrode processes are associated with the peak at 0.35 V and could be reverted by shifting the potential back to 0.1 V or to more negative values (Figure 2d). The phosphate adlayer on Au(111) was also found to be disordered in a pH 4 medium.^32^

#### 3.2.1 AAUA-Modified Au(111) Electrode in 0.1 M PBS

**Figure 3:**
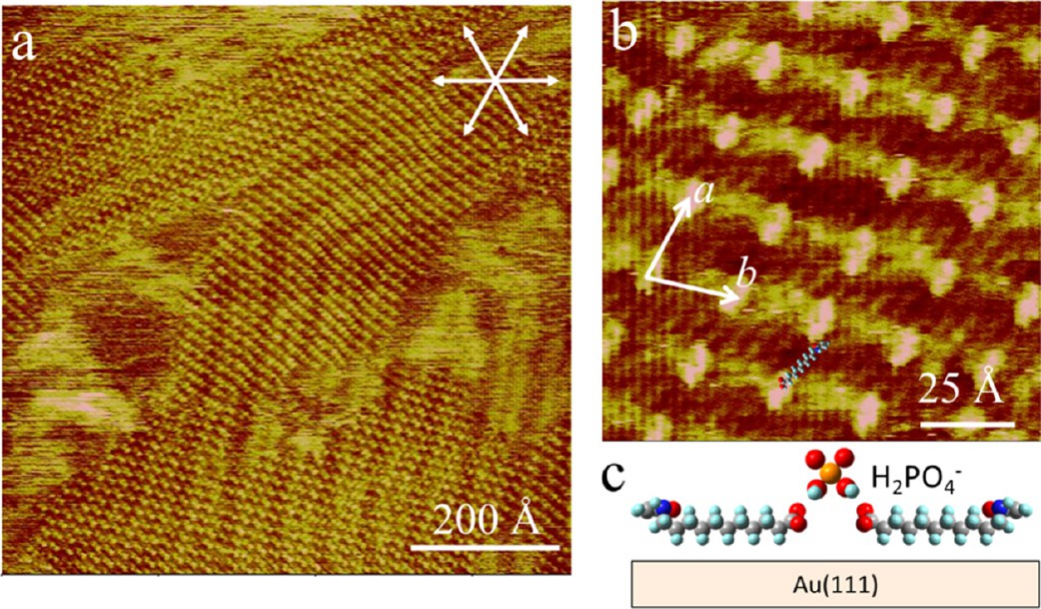
*In situ* STM images showing the typical surface morphology (a) and molecular structures (b) of the AAUA-coated Au(111) electrode at −0.15 V in 0.1 M PBS. Panel (c) shows the side view of the H_2_PO_4_^–^-bridged AAUA linkage. Imaging condition: *V*_b_ = −200, *I*_t_ = 0.8 nA.

The STM results obtained with the AAUA-modified Au(111) electrode in 0.1 M PBS are now described. Starting at −0.15 V, the gross-scale STM scan shown in Figure 3a reveals the typical surface structure on this sample, where elongated linear patterns are superimposed on the typical reconstructed Au(111) surface. The linear features in the background are aligned in the ⟨121⟩ axis of the Au(111) surface and repeat in 64 Å laterally across the surface. This is the reconstructed Au(111)-(√3 × 22) phase,^33^ which was not affected by the AAUA’s adsorption. A few patches of reconstructed Au(111) appear to be AAUA-free. They are brighter than the AAUA-occupied domains.

Scanning with −200 mV bias voltage and 0.8 nA set point current, we obtained a finer resolution STM image (Figure 3b), enabling a clear view of the internal structure of the superimposed adlattice seen at −0.15 V. This two-dimensional (2D) ordered grid pattern is pseudosquare, defined by two vectors, *a* and *b*, 0 and 14° off the ⟨110⟩ axis of the Au(111) substrate. They are both 15 ± 0.5 Å long and enclose an angle of 74°. Linear features 0.4 Å lower than the corner spots were seen in the background. They are ascribed to the alkane chains of the AAUA admolecules.

It is stressed that this grid structure was observed only in PBS, not in K_2_SO_4_ (see below), implying that anion (HPO_4_^2–^ or H_2_PO_4_^–^) was coadsorbed with AAUA and is associated with the bright spots located at the corners of the grid pattern (Figure 3b). Since the −COOH group of the AAUA admolecule has a p*K*_a_ value of 3.3, it likely dissociated to −COO^–^ in the neutral PBS, which enabled hydrogen bonding with an H_2_PO_4_^–^ anion, as illustrated by the ball model depicted in Figure 3c. This interaction could raise the −COO^–^ groups and shortened the lateral dimension of the AAUA admolecule from the ideal 18 to the measured 15 Å on the Au electrode. This grid was stable against protracted scanning for more than 2 h. This interfacial H_2_PO_4_^–^ anion was not adsorbed directly on the bare Au(111) at −0.15 V but lay at a higher plane than the adsorbed AAUA.

Moreover, the importance of the interaction between coadsorbed H_2_PO_4_^–^ anions and the −COO^–^ groups of the AAUA admolecules *via* hydrogen bonds was substantiated by conducting STM imaging of an Au(111) sample premodified with *N*-dodecylacrylamide (DDA) in 0.1 M PBS. Note that DDA has a molecular structure similar to that of AAUA but without the – COOH group. The DDA molecular adlayer prepared by the same method was highly ordered on the reconstructed Au(111) surface (shown in the Supporting Information, Figure S1). DDA molecules arranged in the typical striped pattern observed in K_2_SO_4_ (shown below). After scanning for 3 h, the grid pattern of AAUA (Figure 3b) was not found with DDA. This result supports our contention that the −COO^–^ of AAUA was important in facilitating the interaction with H_2_PO_4_^–^ in PBS.

**Figure 4:**
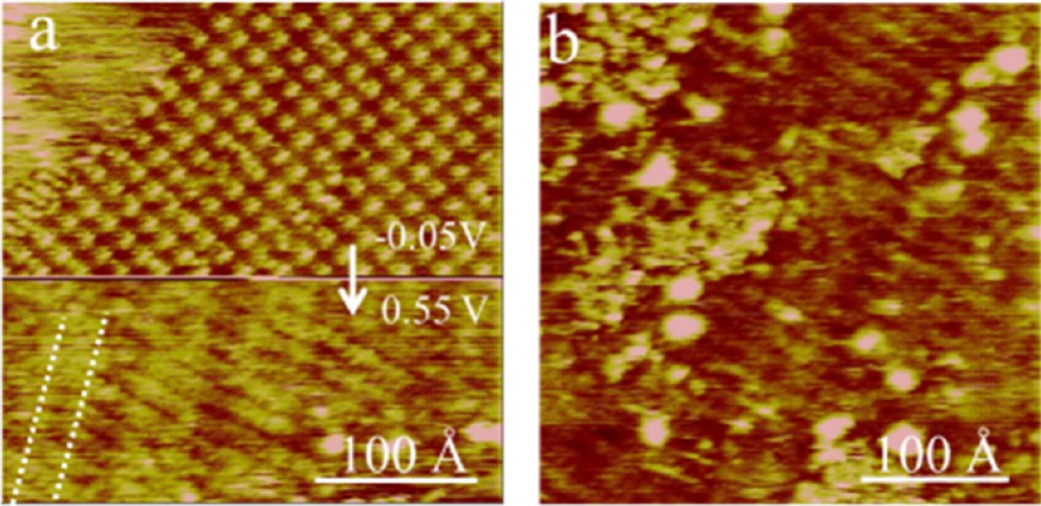
*In situ* STM images showing the restructuring of the AAUA adlayer on Au(111) in response to an abrupt potential step from −0.05 to 0.55 V in the middle of a downward scan (a). The next upward scan shown in panel (b) was collected 1 min after (a). Dotted lines marked in panel (a) show the reconstructed Au(111). The tunneling conditions are −100 mV in base voltage and 1 nA in tunneling current.

In order to scrutinize the electrode process associated with peak A in the CV profile (Figure 1b), we switched the potential abruptly from −0.05 to 0.55 V in the middle of a downward scan. A composite STM image was collected (Figure 4a), which shows that the AAUA adlayer restructured instantaneously from the grid to a striped pattern, seen in the upper and lower half of Figure 4a, respectively. The reconstructed Au(111) surface remained intact (marked by the dotted lines). This scan was acquired in 12.6 s with a scan rate of 20.35 Hz with a 512 line/frame resolution.

The immediate following upward scan was collected (Figure 4b), revealing only a faint linear pattern in the background and poorly defined decorations of protrusions. In line with the CV result (Figure 1b), the coadsorption of H_2_PO_4_^–^ or HPO_4_^2–^ is expected at 0.5 V. These presumed blotches grew rapidly to the entire Au electrode with a more positive potential, resulting in a roughened Au electrode within 20 min. The subsequent imaging effort shows that these anions could displace some AAUA admolecules from the Au electrode.

**Figure 5:**
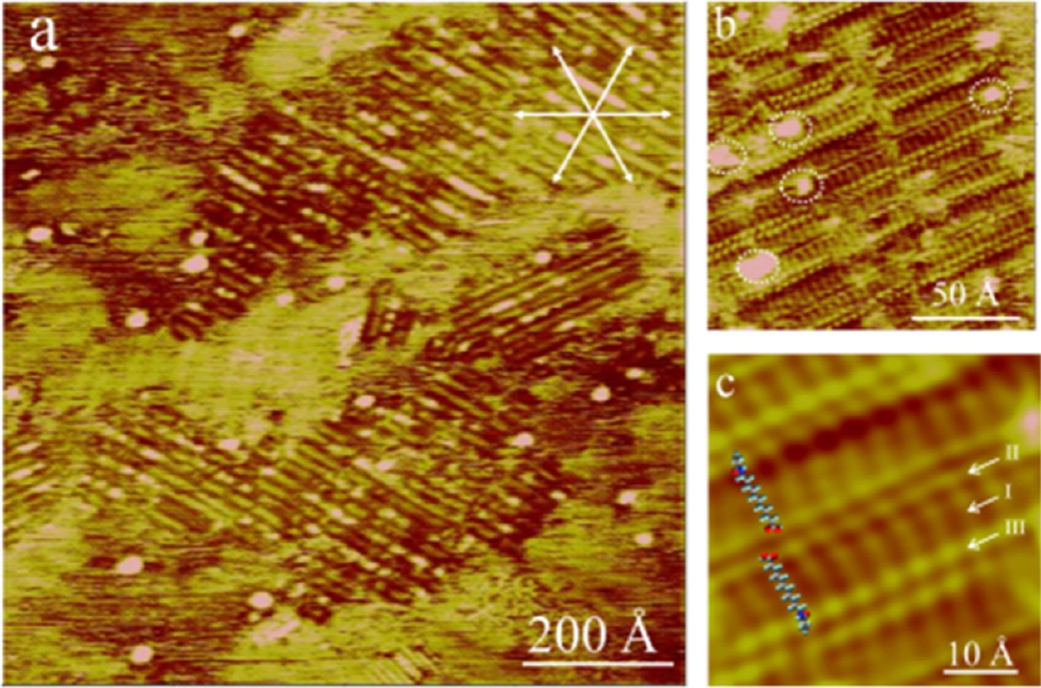
*In situ* STM images showing AAUA stripes on Au(111) after the potential was shifted from 0.55 to −0.05 V (a). High-resolution STM images (b, c) reveal the internal structures of the striped AAUA phase. Dotted circles marked in panel (b) highlight coadsorbed anions. The internal functionalities of −C_10_, −COO^–^, and acrylamido of AAUA admolecules are marked in parts I, II, and III in (c). The tunneling conditions are −250 mV in base voltage and 1 nA in tunneling current. AAUA admolecules in neighboring lamellae orient with the −COO^–^ (or acrylamide) groups facing each other, as illustrated by the molecular models.

If the potential was held at 0.55 V briefly (∼2 min) before it was stepped to −0.05 V, one would have the AAUA stripe back on the reconstructed Au(111) electrode, as shown in Figure 5a. The internal structure of the AAUA stripe is revealed by the high-resolution STM scans shown in Figure 5b,c. Distinct rows of circular protrusions and connecting linear segments are noted. The linear segment marked by **I** ∼10 Å long are readily associated with the C_10_ alkane chains of AAUA admolecules. They are equally separated by 4.8 Å (center-to-center) within a lamella aligned parallel to the ⟨121⟩ direction of the Au(111) substrate. The weak and strong protrusions in rows **II** and **III** are ascribed to −COO^–^ and acrylamido groups of AAUA, respectively.

The adsorbed H_2_PO_4_^–^ anions seen at 0.55 V were not completely desorbed at −0.05 V, which were imaged as bright spots, marked by dotted circles in Figure 5b. A close examination of Figure 5b reveals that these randomly dispersed spots are mostly located near the presumed −COO^–^ sites of the AAUA molecules. All AAUA molecules are aligned perpendicularly to the lamella direction, with their alkane backbones running parallel to the ⟨110⟩ axis of the Au(111) surface. The acrylamide and acidic groups of AAUA in two neighboring lamella arrange face-to-face, as illustrated by the molecular models superimposed on the STM image in Figure 5c.

The dimension of the AAUA admolecule measured from its centers of the acrylamide and acidic groups is only 11 Å, much smaller than the ideal 18 Å for a fully stretched AAUA. This result suggests that AAUA admolecules oriented in a way that their acrylamide and acidic groups were tilted away from the Au electrode. This structure might be driven by the adsorption of H_2_PO_4_^–^ hydrogen bonded to two −COO^–^ groups, as mentioned above (Figure 3b). This spatial arrangement also has hydrophobic acryl groups close to each other.

Therefore, the ordered AAUA structure was restored by changing the potential to the negative region after a momentary pause at 0.55 V. However, if the potential was held at 0.5 V for 30 min and then stepped back to −0.2 V, the AAUA stripe was partly restored (<50%). It appears that the adsorption of anion became so important that some AAUA admolecules were displaced and possibly dissolved in the solution phase at *E* > 0.5 V. These lost AAUA molecules might not be recaptured by the Au electrode, and the pristine AAUA grid structure was not retrieved when the potential was made negative to −0.05 V again.

#### 3.2.2 AAUA/Au(111) Interface in 0.1 M K_2_SO_4_

**Figure 6:**
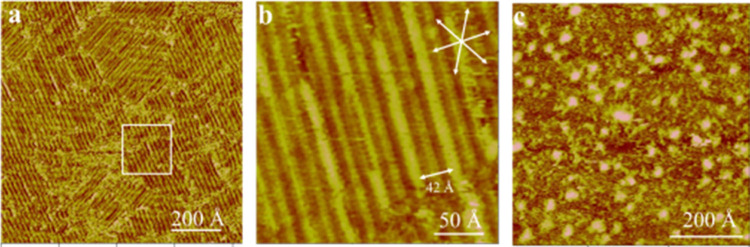
*In situ* STM images showing the texture (a) and internal structure (b) of the AAUA monolayer supported by Au(111) at 0.1 V in 0.1 M K_2_SO_4_. Panel (c) shows the sample surface after the potential was changed to 0.6 V.

The AAUA-modified Au(111) sample was also examined in 0.1 M K_2_SO_4_ to reveal the substantial role of anions in building the interfacial structure. The sample, prepared by immersion in 10 mM AAUA/methanol for 1 min, has prominent linear patterns arranged in ∼250 Å patches, as revealed by the STM image shown in Figure 6a. As with PBS, the adsorbed AAUA molecule did not alter, but indeed obscured, the reconstructed Au(111) structure when imaging at −200 mV bias voltage and 1 nA feedback current.

The internal structure in a linear patch was seen with a finer resolution STM scan, as shown in Figure 6b, revealing elongated lines running in the ⟨121⟩ direction of the Au(111) electrode. This contrasts markedly with the grid pattern seen in PBS (Figure 3b), indicating the presence of anions on the AAUA-modified Au(111) electrode.

Shifting the potential from 0.1 to 0.6 V quickly removed the AAUA stripe and resulted in a rough Au sample surface. The reconstructed Au(111) surface was also lifted to the (1 × 1) phase, producing protruding Au islands (Δ*z* = 2.3 Å) seen in Figure 6c. In line with the reasoning for PBS, the drastic changes of the AAUA/Au(111) interface at 0.6 V are assumed to derive from the prominent anion adsorption and dissolution of AAUA admolecules.

**Figure 7:**
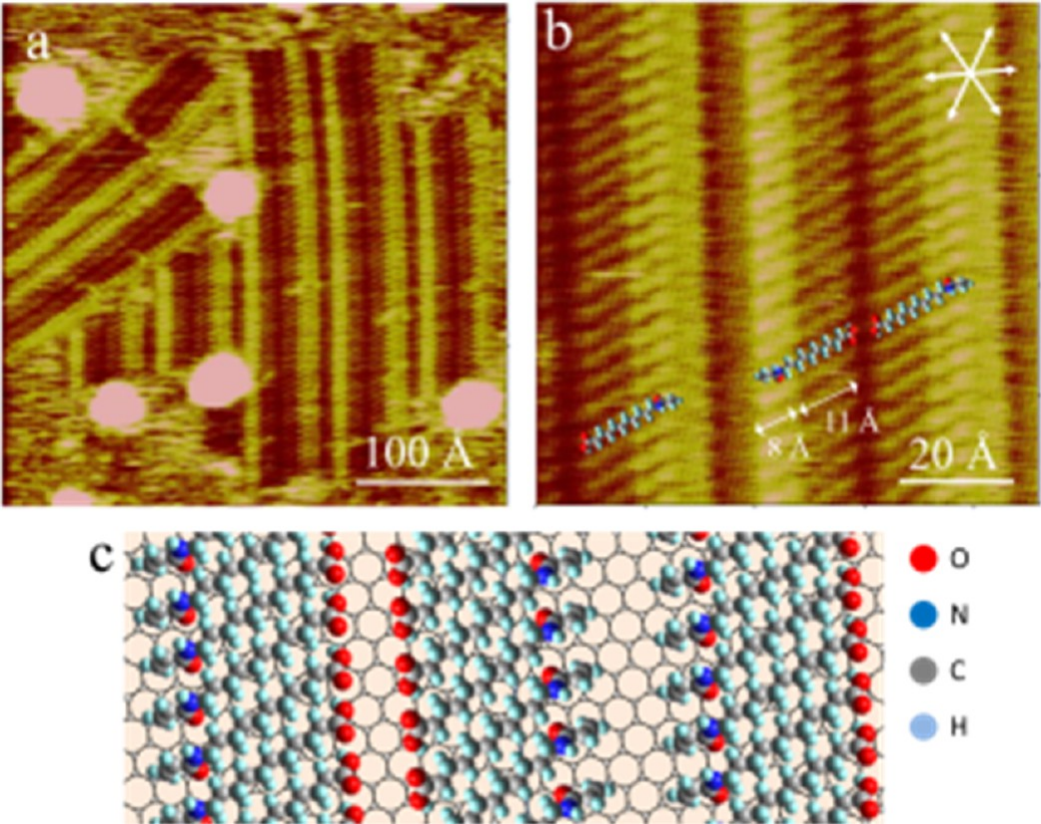
*In situ* molecular-resolution STM images showing two rotational domains (a) and the internal structure (b) of the striped AAUA on the Au(111) electrode at 0.3 V in 0.1 M K_2_SO_4_. Panel (c) shows the molecular model of this AAUA striped phase.

To probe the reversibility of this restructuring event, we then switched the potential from 0.6 to 0.3 V. This act quickly restored the AAUA stripes (<10 min) in local areas, as seen in Figure 7a. Two linear patches are seen, which enclose a 60° angle. They are preferentially aligned in the ⟨121⟩ direction of Au(111). The quality of Figure 7a is good enough to ensure that the Au(111) substrate still assumed the (1 × 1) phase. The ordered AAUA striped pattern could thus be formed on both the reconstructed and (1 × 1) phases of Au(111).

The insight of the AAUA striped pattern could be seen with the finer resolution STM scan shown in Figure 7b, where linear segments are stacked vertically or in the ⟨121⟩ direction of the Au(111) surface. Each linear segment comprises two corrugated sections (0.5 Å difference) when tunneling at −300 mV in bias voltage and 0.8 nA in feedback current. The dimer linear segment ∼14 Å long is ascribed to the −COO^–^ + C_10_, whereas the bright section ∼8 Å long corresponds to the acrylamido group of the AAUA molecule. These results indicate AAUA admolecules were adsorbed horizontally on the Au(111) electrode.

The molecular axes of AAUA admolecules are oriented 60° to the stripe’s direction or parallel to the ⟨121⟩ axis of the Au(111) electrode, as opposed to the ⟨110⟩ direction seen in PBS. Three AAUA molecular models are depicted in Figure 7b to illustrate the acid-to-acid (acrylamide-to-acrylamido) spatial arrangement between two neighboring lamellae. This feature is presumed to result from the hydrophilic and hydrophobic natures of these functional groups of the AAUA molecule. The nearest neighbor spacing within a lamella is 4.8 ± 0.2 Å.

**Figure 8:**
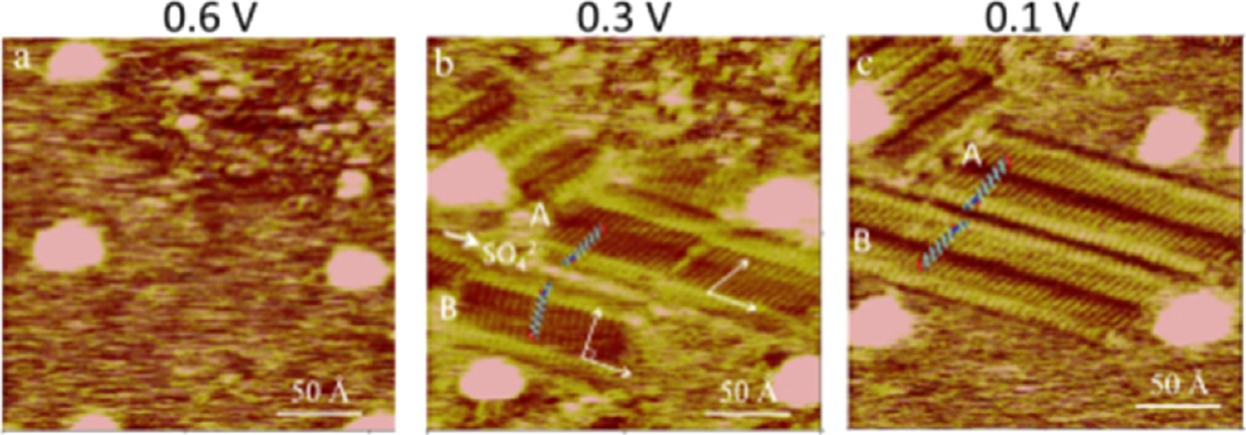
*In situ* STM images showing restructuring of the AAUA molecules adsorbed on the Au(111) electrode, as the potential was changed from 0.6 (a) to 0.3 (b) and 0.1 (c) in 0.1 M K_2_SO_4_. The tip potential was fixed at −0.2 V, and the feedback current was 0.8 nA. Stripes **A** and **B** consist of AAUA admolecules oriented differently in panel (b) but equally in panel (c). The protruding line sandwiched between **A** and **B** is attributed to adsorbed SO_4_^2–^.

More insight into the dynamics of the electrode process driven by potential stepping could be gained from real-time STM imaging. The AAUA-modified Au(111) sample was first imaged at 0.6 V, more positive than the A2 peak (Figure 1c), where AAUA and SO_4_^2–^ anions were coadsorbed on an apparently rough Au electrode, as seen in Figure 8a. Shifting the potential from 0.6 to 0.3 V (negative of A2) restored local AAUA stripes within 1 min, which grew from 50 to 300 Å within 2 min scanning at 0.3 V (Figure 8b). A bright line (pointed by an arrow in Figure 8b) is also noted between two AAUA stripes, marked **A** and **B**.

As the potential was shifted from 0.3 to 0.1 V, the bright line highlighted in Figure 8b disappeared. This implies that this protruding line was adsorbed SO_4_^2–^ anions, which were drawn to the Au electrode by the positive potential and were repelled by the negative potential. Their interaction with the Au electrode was strong enough to displace some AAUA admolecules and contacted with the Au electrode. Adsorbed sulfate anions might not interact specifically with the AAUA admolecules. The stable AAUA stripe pattern seen at 0.1 V is revealed in Figure 8c, which has the same striped appearance as that seen in Figure 7b but is rotated 60°.

Figure 8a–c was collected at the same spot on the Au electrode, as indicated by the protruding Au islands at the four corners of the array. A few details in the AAUA structure are seen in this sequence of STM images. First, at 0.3 V, two azimuthal orientations of AAUA admolecules are noted in Figure 8b. Those in the **A** and **B** stripes enclose angles of 60 and 90° with the stripe direction, respectively. However, those in stripe B convert to the 60° configuration, as the adsorbed SO_4_^2–^ anions came off the Au electrode at 0.1 V (Figure 8c). This process is registered as the A1/C1 peak in the CV profile (Figure 1c). Therefore, the adsorption configuration and spatial structure of the AAUA molecule on the Au electrode were affected by the applied potential and coadsorbed anions. A close examination of these images reveals that the presumed SO_4_^2–^ anions appeared at sites near the acrylamide groups, not the −COO^–^ sites of AAUA admolecules, as anticipated from the negative charges of these entities.

This AAUA striped structure resembles those found with a number of alkane molecules adsorbed on Au(111).^11,12^ Compared with these alkane molecules, AAUA has a more complicated molecular structure and more versatile intermolecular interactions. In particular, in addition to the most common van der Waals interaction, the acidic and acrylamide groups of AAUA can form hydrogen bonds with neighbors, regulating them into stacked lamella. These intermolecular forces work together to enable the efficient self-assembly of AAUA into an ordered array on the Au(111) electrode. This was completed with a 1 min immersion in a 0.1 mM AAUA solution.

It is fair to state that the hydrophilic and hydrophobic natures of the functional groups in an organic molecule are important to guide its azimuthal orientation on a substrate. This view can serve as one of the guidelines used to design molecular organization on a chemically modified electrode. The potential control provides another means to manipulate the adsorption of organic molecules in an electrochemical environment. The drastic change of the spatial structure of AAUA with potential modulation implies a notable change of AAUA’s adsorption configuration. In particular, the −COO^–^ end could be forced to bind with the Au electrode, leading to an upright orientation at positive potential. However, this study has not resulted in an ordered upright AAUA structure on the Au electrode. This could be realized by increasing the AAUA dosing concentration and changing the nature of the interface by replacing the electrolyte.

## 4 Conclusions

*In situ* STM imaging has revealed the organization of AAUA molecules adsorbed on the Au(111) electrode under potential control in PBS and K_2_SO_4_. Thanks to the van der Waals force between the alkyl groups and hydrogen bonds formed between two −COOH and two −NHCO– groups of AAUA molecules, the adsorption and subsequent assembly processes of AAUA molecules into ordered lamellar arrays on the Au(111) surface were efficient in a methanol medium. The −COOH group of AAUA admolecule likely dissociated in the neutral PBS and K_2_SO_4_, producing −COO^–^ group facilitating hydrogen bonds with H_2_PO_4_^–^ anions and the formation of a unique grid structure in PBS. By contrast, only the lamella structure was found in K_2_SO_4_, whose characteristics (azimuthal orientation and lateral width) differed from those seen in PBS. These results reflect the profound effect of interaction between anion (phosphate and sulfate) and AAUA admolecules on the spatial structure of AAUA on the Au electrode. As with bare Au(111), the extent of anion adsorption increased at a more positive potential on the AAUA-modified Au(111) electrode, resulting in the displacement of AAUA admolecules and roughening of the interface. This loss of AAUA admolecules at positive potential made it impossible to restore the pristine ordered AAUA adlayer when the potential of the Au electrode was made negative again.

## Supporting Information Available

*In situ* STM images acquired with Au(111) premodified with *N*-dodecylacrylamide (DDA) in 0.1 M PBS (PDF)
