# Supplementary material for: Effects of Anion Coadsorption on the Self-Assembly of 11-Acryloylamino Undecanoic Acid on an Au(111) Electrode
Source: ACS Omega. 2024 Sep 10;9(38):39827–35. doi: 10.1021/acsomega.4c05080 (PMC11425958; doi:10.1021/acsomega.4c05080)
Supplement: Supplementary file 1 — ao4c05080_si_001.pdf [file ao4c05080_si_001.pdf]

Supporting information

The Effects of Anion Coadsorption on the Self-Assembly of 11-acryloylamino Undecanoic Acid on Au(111) Electrode

Yi-Ting Huang,[a] Jia-Yin Chen,[b] Chiao-An Hsieh,[a] Yamuna Ezhumalai,[a] Chun-Jen Huang[b,c,]* and Shuehlin Yau[a,]*

[a]Department of Chemistry, National Central University, Chungli County, Taoyuan City, Taiwan 32049, ROC

[b]Department of Chemical and Materials Engineering, National Central University, Chungli County, Taoyuan City, Taiwan 32049, ROC

[c]R&D Center for Membrane Technology, Chung Yuan Christian University, 200 Chung Pei Rd., Chung-Li City 32023, Taiwan.

Corresponding authors:

*Shuehlin Yau (Email: yau6017@ncu.edu.tw)

*Chun-Jen Huang (Email: cjhuang@ncu.edu.tw)

Submitted to *ACS Omega*

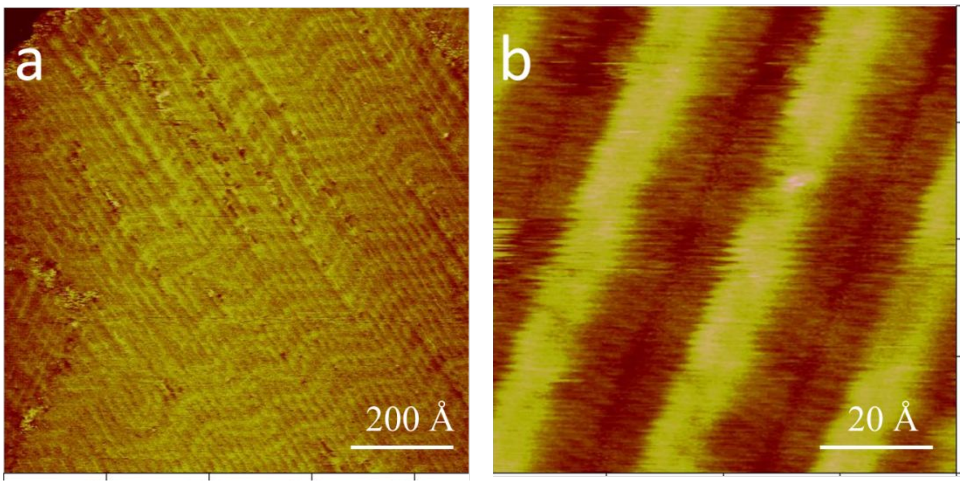

Figure S1. In situ STM images showing the smooth morphology (a) and long-range ordered N-Dodecylacrylamide (DDA) adlayer (b) on the Au(111) electrode at -0.15 V in 0.1 M PBS.
